# Peripheral memory B cells in multiple sclerosis vs. double negative B cells in neuromyelitis optica spectrum disorder: disease driving B cell subsets during CNS inflammation

**DOI:** 10.3389/fncel.2024.1337339

**Published:** 2024-02-07

**Authors:** M. P. Tieck, N. Vasilenko, C. Ruschil, M. C. Kowarik

**Affiliations:** Department of Neurology and Stroke, Center for Neurology, and Hertie-Institute for Clinical Brain Research Eberhard-Karls University of Tübingen, Tübingen, Germany

**Keywords:** neuromyelitis optica spectrum disorder, NMOSD, multiple sclerosis, DN B cells, memory B cells, AQP4-antibodies, EBV

## Abstract

B cells are fundamental players in the pathophysiology of autoimmune diseases of the central nervous system, such as multiple sclerosis (MS) and neuromyelitis optica spectrum disorder (NMOSD). A deeper understanding of disease-specific B cell functions has led to the differentiation of both diseases and the development of different treatment strategies. While NMOSD is strongly associated with pathogenic anti-AQP4 IgG antibodies and proinflammatory cytokine pathways, no valid autoantibodies have been identified in MS yet, apart from certain antigen targets that require further evaluation. Although both diseases can be effectively treated with B cell depleting therapies, there are distinct differences in the peripheral B cell subsets that influence CNS inflammation. An increased peripheral blood double negative B cells (DN B cells) and plasmablast populations has been demonstrated in NMOSD, but not consistently in MS patients. Furthermore, DN B cells are also elevated in rheumatic diseases and other autoimmune entities such as myasthenia gravis and Guillain-Barré syndrome, providing indirect evidence for a possible involvement of DN B cells in other autoantibody-mediated diseases. In MS, the peripheral memory B cell pool is affected by many treatments, providing indirect evidence for the involvement of memory B cells in MS pathophysiology. Moreover, it must be considered that an important effector function of B cells in MS may be the presentation of antigens to peripheral immune cells, including T cells, since B cells have been shown to be able to recirculate in the periphery after encountering CNS antigens. In conclusion, there are clear differences in the composition of B cell populations in MS and NMOSD and treatment strategies differ, with the exception of broad B cell depletion. This review provides a detailed overview of the role of different B cell subsets in MS and NMOSD and their implications for treatment options. Specifically targeting DN B cells and plasmablasts in NMOSD as opposed to memory B cells in MS may result in more precise B cell therapies for both diseases.

## Introduction

B cells have been shown to play a key role in the pathogenesis of several autoimmune diseases. The main functions of B cells are antigen recognition and specific antibody production, as well as antigen presentation and cytokine secretion (Cyster and Allen, 2019). In autoimmune diseases, a major function of B cells can be recognition of self-antigens, possibly by escaping self-tolerance and/or molecular mimicry, and production of auto-reactive antibodies (Hampe, 2012; Bonasia et al., 2021). The secretion of antibodies can be detrimental in certain neuro-immunological diseases exemplified by myasthenia gravis, neuromyelitis optica spectrum disorder (NMOSD), MOG antibody associated disorder (MOGAD) or LGI1/NMDA receptor encephalitis (Prüss, 2021). During these autoimmune diseases, auto-antibodies either interfere with the function of the molecules they recognize [e.g., acetylcholine receptor antibodies (AChR) in myasthenia gravis] and/or lead to cell destruction by complement-dependent cytotoxicity (CDC) (e.g., NMOSD) or antibody-dependent cell mediated cytotoxicity (ADCC). Furthermore, B cells can function as antigen-presenting cells, potentially triggering a pathological immune response involving T cells. The secretion of pro-inflammatory cytokines such as IL6, TNFα or interferon gamma might further support autoimmune reaction in terms of a pro-inflammatory milieu and stimulation of immune cells.

This review contrasts the differences in the pathophysiology of MS and NMOSD, with a particular focus on peripheral B cells and B cell subsets and their association with CNS B cells and inflammation. Besides the overall differences of B cell subsets in disease pathology, we summarize the effects of disease-specific treatments on B cell populations and their implications for the pathophysiology of both diseases. Additionally, we explore potential B cell subset-specific treatments for future therapies. The mechanism of action of each drug discussed throughout the text is summarized in Table 1. An overview of B cell surface makers is provided in Table 2.

**TABLE 1:** B cell subset alterations in peripheral blood under various treatments.

	Mode of action	Lymphocyte count	Total B cells	% Naive B cells	% Memory B cells	% Bregs	% Plasmablast	% DN	References
Cladribine	Purine nucleoside analog selectively depleting peripheral lymphocytes through inhibition of enzymes involved in DNA metabolism	↓	↓	↑	↓	–	↔	↔	Ceronie et al., 2018; Ruschil et al., 2023
Teriflunomide	Reversibly inhibits dihydro-orotate dehydrogenase. Reduction in proliferation of activated T and B lymphocytes without causing cell death	↓	↓	–	↓	–	↓	–	Yilmaz et al., 2021
Mitoxantrone	Type II topoisomerase inhibitor. Disrupts deoxyribonucleic acid synthesis and repair	↓	↓	↑	↓	–	–	–	Kim et al., 2011
Fingolimod	Sphingosine-1-phosphate receptor modulator	↓	↓	↓	↓	↑	↓	↑	Blumenfeld-Kan et al., 2019; Kemmerer et al., 2020; Kowarik et al., 2021
Siponimod	Sphingosine-1-phosphate receptor modulator	↓	↓	↓	↔	↑	–	–	Wu et al., 2020
Natalizumab	Humanized monoclonal antibody against the cell adhesion molecule α4-integrin	↑	↑	↑	↑	–	↓		Kemmerer et al., 2020; Kowarik et al., 2021
Alemtuzumab	Monoclonal antibody against CD52	↓	↓↑	–	–	–	–	–	Baker et al., 2017a,b
Ocrelizumab	Monoclonal antibody against CD20	↓	↓	↓	↓	–	↓	↓	Hauser et al., 2021
Interferon Beta	Inhibition of T-cell activation and proliferation, apoptosis of autoreactive T cells, induction of regulatory T cells, inhibition of leukocyte migration across the blood-brain barrier.	↔	↔	↑	↑	–	NC	NC	Kemmerer et al., 2020
Dimethyl fumarate	Interfere with the aerobic glycolysis of activated lymphoid cells with a high metabolic turnover	↓	↓	↑	↓	↔	↔	↓	Spencer et al., 2015; Smith et al., 2017; Kemmerer et al., 2020
Glatiramer acetate	Inhibits the T cell response to several myelin antigens	↔	(↓)	↔	(↑)	–	NC	NC	Kemmerer et al., 2020
Rituximab	Monoclonal antibody against CD20	↓	↓	↓	↓	↑	↓	↑	Quan et al., 2015; Ramwadhdoebe et al., 2019
Ofatumumab	Monoclonal antibody against CD20	↓	↓	–	–	–	–	–	Bar-Or et al., 2022
Inebilizumab	Monoclonal antibody against CD19	↓	↓	↓	↓	–	↓	↓	Agius et al., 2019
Tocilizumab	Monoclonal antibody against interleukin-6 receptor	↔	↔	↑	↓	–	↓	↓	Liu et al., 2021
Eculizumab*	Monoclonal antibody against complement C5	–	–	–	↓	–	–	–	Li et al., 2021b

NC: no clear changes. The arrows in parenthesis mean a slight change. DN, double negative; Bregs, regulatory B cells. The (-) means no data found. *In patients with myasthenia gravis.

**TABLE 2:** Overview of B cell surface makers.

	Transitional B cells/Regulatory B cells	Naïve B cells	Memory B cells	Plasmablasts	DN B cells
IgD	+	+	−	−	−
CD 19	+	+	+	+	+
CD 20	+	+	+	Low	Low
CD 27	−	−	+	+	−
CD 38	+	Low	−	High	Low
CD 5	+	−	−	/	/
CD 9	+	/	/	/	/
CD 24	+	Low	Low	/	/
CXCR5	/	+	+	+	±

IgD, immunoglobulin D; CD, cluster of differentiation; DN, double negative. “/” means unknown data.

### B cell subsets and development

B cells undergo several stages of maturation during adaptive immune responses. Shortly summarized, B cells are generated in the bone marrow (pre/pro B cells) and then released into the peripheral blood (Cyster and Allen, 2019). Naïve B cells (antigen inexperienced) then migrate from the peripheral blood to secondary lymphoid tissues such as the spleen or lymph nodes, where they undergo further differentiation in germinal center reactions (Kurosaki et al., 2015; Cyster and Allen, 2019). Once bound to an antigen, B cells undergo a series of receptor and Ig subclass expression changes with co-stimulatory signaling by, e.g., T cell help. After this differentiation process, different subsets of antigen-experienced B cells emerge: memory B cells and plasmablasts. When memory B cells reencounter specific antigens, they undergo expansion and differentiate into plasmablasts mostly in the germinal centers (Kurosaki et al., 2015). Plasmablasts develop subsequently in either short-lived plasma cells or long-lived plasma cells which can maintain antibody production for decades without antigen re-stimulation (Lightman et al., 2019).

In contrast, another heterogeneous B cell group has the ability to suppress immune responses and are named regulatory B cells as a functionally defined population (Catalán et al., 2021). A definitive set of phenotypic markers are still lacking (Catalán et al., 2021). IL-10, IL-35, and TGF-beta secretion, the cell surface proteins CD1d and PD-L1 characterize the anti-inflammatory properties of this cell group (Catalán et al., 2021). Immature transitional B cells, divided into T1, T2, and T3 subpopulations are an intermediate stage between immature cells from the bone marrow and mature cells in the periphery (Catalán et al., 2021). T1 and T2 subtypes constitute a significant source of functional regulatory B cells (Zhou et al., 2020; Catalán et al., 2021). Autoimmune diseases are prone to have a lower frequency of regulatory B cells (Zhou et al., 2020).

Double negative (DN) B cells constitute another B cell population that lacks expression of immunoglobulin D and CD27 surface markers and has shown to be associated with autoimmune diseases (Sanz et al., 2019; Ruschil et al., 2021). The class-switched IgD- phenotype may indicate an antigen-specific maturation. Some author suggests that in the absence of CD27, a transition from a naive B cell seems unlikely (Li et al., 2021a). However, transcriptome analysis points toward a continuum of naive B-cells, memory B cells and plasmablasts (Ruschil et al., 2020), although the exact origin and maturation pathway of DN B cells is still unclear. Jenks et al. (2018) found two subgroups of DN B cells mainly based on the expression of the follicular marker CXCR5 (DN1: CXCL5 + and DN2: CXCL5- subtype), which is involved in the migration of B cells into B-cell follicles. CXCL5 + DN B cells are mostly expanded in elderly healthy individuals, while CXCL5- DN B cells were markedly found in active systemic lupus erythematosus (SLE), a defined autoantibody associated disease. In SLE, it has been further shown that the CXCL5- DN B cell subset develops from an activated naïve B cell pool. The lack of CXCR5 point toward an extrafollicular maturation pathway (Tipton et al., 2015; Jenks et al., 2018; Sanz et al., 2019). Compelling evidence suggests that CXCL5- DN B cell subset represents a primed precursor population for antibody-secreting cells (Jenks et al., 2018; Sanz et al., 2019). Although our studies didn’t differentiate these two DN B cell subpopulations, we have also shown that DN B cells are as well up-regulated in various auto-inflammatory neurological diseases including myasthenia gravis, Guillain-Barreì syndrome and NMOSD but not consistently in MS (Ruschil et al., 2020). We and others could show that this population most likely represents a transient precursor B cell population undergoing differentiation into antibody-secreting cells (Ruschil et al., 2020). Along these lines, we could show that the proportion of peripheral DN B cells is increased after vaccination and DN B cell-derived recombinant antibodies showed binding of specific vaccines, providing indirect evidence of their antibody secretion capacity (Ruschil et al., 2020).

### Pathophysiological roles of B cells in autoimmunity

The development of auto-reactive properties of B cells in autoimmune diseases remains a topic of great debate. While molecular mimicry is a recognized mechanism that can mislead B cells toward self-antigens, impaired self-tolerance during B cell development also contributes to auto-reactivity. The initial B cell repertoire generated by random V(D)J recombination undergoes a bimodal removal of autoreactive clones due to exposure to self-antigens (Meffre and O’Connor, 2019). This exposure occurs initially in the bone marrow, the site of B cell generation, and later in the periphery when B cells encounter a new set of self-antigens, resulting in the removal of autoreactive clones (Goodnow, 1996; Wardemann et al., 2003; Meffre and O’Connor, 2019). Distinct mouse models have shown that developing self-reacting B cells can be silenced through the following mechanisms: (1) clonal deletion; (2) clonal unresponsiveness to antigen or anergy; and (3) “receptor editing” or antigen receptor gene replacement by continued V(D)J recombination (Meffre, 2011; Stoehr et al., 2011; Meffre and O’Connor, 2019). Several lines of evidence suggest that central tolerance is likely dysregulated in NMOSD (Cotzomi et al., 2019; Meffre and O’Connor, 2019). The identification of pathogenic anti-AQP4 clones, which originate from unmutated autoreactive naive B cells in patients with NMOSD, is in potential agreement with this scenario (Meffre and O’Connor, 2019). In contrast, MS patients exhibit distinct B cell tolerance patterns compared to other autoimmune diseases. Here, an impaired peripheral B cell tolerance checkpoint is believed to be the main culprit, leading to the peripheral buildup of polyreactive mature naïve B cells, as shown by Kinnunen et al. (2013). Consistent with this assumption, regulatory T cells (Tregs) in MS patients seem to exhibit impaired suppressive activity and abnormally secrete interferon gamma (IFNγ) (Dominguez-Villar et al., 2011).

Molecular mimicry arises when peptides from pathogens display structural similarities with self-antigens. The presence of diverse pathogens, with each having its own potential unique molecular mimic to a CNS antigen, may elucidate why researchers have struggled to link a specific virus to, e.g., multiple sclerosis (Libbey and Fujinami, 2014). However, Epstein-Barr virus (EBV) has been identified as a potential viral agent that may trigger the production of autoreactive antibodies targeting GlialCam (Lanz et al., 2022). Nonetheless, more extensive evaluation of these findings is required. To the best of our knowledge, there is presently no conclusive evidence of established pathogens incorporating molecular mimicry mechanisms in NMOSD.

Regarding other mechanisms of B cell-mediated autoimmunity, B cells contribute to the development of diabetes through recognition of self-antigens with autoreactive antibodies and presentation of self-antigens via MHC class II molecules to T cells (Serreze et al., 1996). These findings indicate that self-antigen presentation by autoreactive B cells, which evade tolerance, could be the catalyst for the onset of autoimmune disorders. In multiple sclerosis, HLA class II alleles of the DR2 haplotype, DRB1*1501, DRB5*0101, and DQB1*0602, are well established genetic risk factors for MS and show a functional redundancy in Ag presentation (Sospedra and Martin, 2006). Thus, B cells serving as antigen presenting cells may shape an autoreactive T cell repertoire by presenting autoantigens by DR2 HLA-DR molecules (Wang et al., 2020).

Finally, altered cytokine levels that may result from a misdirected B cell activation can provide a pathogenic milieu for autoimmunity. Shortly summarized, serum IL-6 concentrations are significantly elevated in patients with NMOSD and are higher than in healthy individuals and patients with MS (Fujihara et al., 2020). Serum cytokine levels in MS do not show a clear proinflammatory profile, and several cytokines have even been shown to be downregulated (Lepennetier et al., 2019; Melamud et al., 2022). Within the CSF compartment, IL6 is also upregulated in NMOSD patients while CXCL13 seems to be a consistently up-regulated B cell-associated cytokine in MS (Sospedra and Martin, 2006). However, B cells are also able to secrete anti-inflammatory cytokines such as IL10 which is also upregulated in the CSF of NMOSD patients (Kaneko et al., 2018).

## B cells in multiple sclerosis

### Evidence for an important role of B cells in MS

With the discovery of oligoclonal bands in the cerebrospinal fluid (CSF) of patients with MS, evidence pointed toward a pathophysiological role of B cells with potentially disease-driving antibodies in the CSF (DiSano et al., 2021). In MS, the majority of B cells in CSF are antigen-experienced B cells (Harp et al., 2007; Eggers et al., 2017), and the frequency of memory B cells is increased in CSF compared to peripheral blood (Eggers et al., 2017). In addition, B cell infiltration has been found within the brain parenchyma (Machado-Santos et al., 2018) and also in leptomeningeal aggregates, which are strongly associated with cortical lesions (Fraussen et al., 2014; Martin et al., 2016; Jain and Yong, 2022). Although Th1/Th17 T cells have at times attracted attention as potential therapeutic targets due to their important role in EAE models, specific CD4, Th1/Th17 immunotherapies have largely failed to show a clear impact on MS relapses (Baker et al., 2017c). In contrast, the high efficacy of B cell depletion in multiple sclerosis, first demonstrated for the CD20-specific B cell depleting agent rituximab (Hauser et al., 2008), was surprising and again highlighted the role of B cells. Subsequent clinical trials with ocrelizumab (Hauser et al., 2017; Vermersch et al., 2022), ofatumumab (Hauser et al., 2020), and ublituximab (Steinman et al., 2022) provide further evidence for the efficacy of (CD20) B cell depletion not only in relapsing but also in primary progressive multiple sclerosis (Kappos et al., 2011; Hauser et al., 2017).

### Possible roles of B cells during MS pathophysiology

**FIGURE 1:**
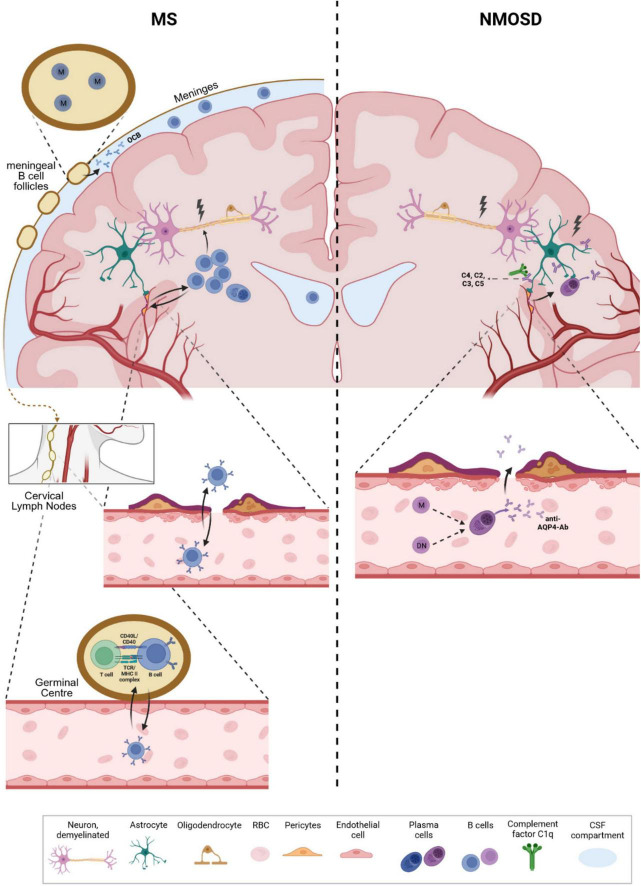
Differential roles of B cells in multiple sclerosis (MS) and neuromyelitis optica spectrum disorder (NMOSD). In MS, B cells have been shown to enter the CNS compartment (via integrin activation) but also recirculate into the periphery by drainage into cervical lymph nodes. B cells show an increased proportion in the CSF, are found in lesions and follicular-like structures at the meninges, and are the source of oligoclonal IgG bands in the CSF of MS patients. Although several interesting antigens have been proposed as potential B cell targets, no clear target or a subset of targets have yet been identified. In addition to antibody secretion, a major function of B cells may be presentation of CNS antigens and stimulation of, e.g., T cells once they have re-entered germinal centers in the periphery. These mechanisms could perpetuate autoimmune cycles leading to recurrent relapses. Memory B cells seem to be of particular interest in this context, although this hypothesis requires further evaluation. In NMOSD, aquaporin-4-specific antibodies are produced in the periphery and target astrocytic end feet, leading to CNS inflammation and breakdown of the blood-brain barrier. During relapses, AQP4-specific B cells are also found in the CSF and may further enhance antibody-mediated, complement-dependent inflammation. Double negative B cells may represent a transient precursor B cell population that differentiates into AQP4-specific antibody-secreting cells through extrafollicular maturation pathways. M: Memory B cells, DN: double negative B cells, OCB: Oligoclonal bands, RBC: red blood cell.

The exact role of B cells in the pathophysiology of MS remains controversial. Epstein-Barr virus (EBV) infections have recently been strongly associated with multiple sclerosis, with 97% of patients in a large cohort showing positive EBV serum titers or seroconversion prior to the development of multiple sclerosis (Bjornevik et al., 2022). In addition, another study suggested molecular mimicry between EBNA1–a prominent EBV antigen–and GlialCAM (glial cell adhesion molecule), suggesting a direct role of pathogenic antibodies in MS (Lanz et al., 2022). However, only a limited number of antibodies reacted against both targets, so further confirmation seems necessary. Other potentially interesting targets for MS antibodies that have been proposed in recent years are chloride-channel protein Anoctamin 2 (ANO2) (Tengvall et al., 2019), which is a transmembrane protein for modulation for neural-excitability; alpha-crystallin B (CRYAB), which is expressed by oligodendrocytes and may have a protective effect by down-regulating the innate immune system (Thomas et al., 2023). Another group recently found antibodies against conformational membrane complexes containing the myelin proteolipid protein 1 (PLP1) (Owens et al., 2023). In addition to these recently described targets, a large number of autoantibodies have been described against various CNS cell types, including neurons, oligodendrocytes and astrocytes, and even immune cells (Fraussen et al., 2014). Although some of these possible antigen-antibody interactions seem to point toward an antibody-driven role of B cells in multiple sclerosis, multiple antigens could not be confirmed in further analyses, and one or a subset of clear antibody targets such as AQP4 in NMOSD have not yet been identified. An alternative function of B cells could be centered around antigen presentation and T cell stimulation. Our group (Kowarik et al., 2021) and other studies (Stern et al., 2014; Ruschil et al., 2021) have demonstrated that B cells not only traverse the blood-brain barrier but also recirculate in the peripheral blood through cervical lymph node drainage (Figure 1.). B cells, potentially primed against antigens in the CNS compartment during relapse, could thus possess the capacity to re-enter germinal centers in the periphery and perpetuate autoimmune circuits (Ruschil et al., 2021). Altogether, it remains unclear whether B cells predominantly produce autoantibodies against specific targets or act as antigen-presenting cells that circulate between the CNS and peripheral compartments; however, the diversity and inconsistency of suggested antigen targets might point toward a substantial antigen-presenting role.

### Peripheral B cells and treatment-specific effects on B cell subsets in MS: consistent effects on memory B cells

Peripheral blood B cell subsets including total B cell numbers, naïve, memory B cells, double negative B cells and plasmablasts during stable disease do not show significant differences when compared to healthy controls (Kemmerer et al., 2020; Ruschil et al., 2020). The prevalence of transitional/regulatory B cells is often low (Zhou et al., 2020) while DN B cells did not show a consistent up-regulation in MS (Fraussen et al., 2019; Ruschil et al., 2020). Besides the broad depletion of circulating B cells by anti-CD20 antibodies such as rituximab, ocrelizumab and ublituximab, several MS treatments have shown to also exert profound effects on peripheral B cells and cerebrospinal fluid (Tables 1, 3). The absolute number of B cells in MS treatments has been shown to be slightly reduced during dimethyl fumarate, fingolimod, and siponimod treatment, unchanged during glatiramer acetate and interferon beta treatment and increased during natalizumab treatment (Kemmerer et al., 2020; Traub et al., 2020). Natalizumab is a monoclonal antibody against the cell adhesion molecule α4-integrin, which is highly expressed in B-cells (Saraste et al., 2016). The increase in peripheral B cell number during natalizumab most likely relies on the egress of memory B cells from the marginal sinus of the spleen through the blockade of integrins by which memory B cells attach to the sinus (Kowarik et al., 2021). However, these cells are also impaired in their ability to cross the blood-brain-barrier so that natalizumab treatment has to be considered separately (Kowarik et al., 2021). Further differential flow cytometric analyses showed, that in most treatments, the fraction of naïve B cells is increased, while the percentage of memory B cells is significantly decreased (Kemmerer et al., 2020; Traub et al., 2020). Regulatory B cells show consistently elevated percentages during most treatments while DN B cells show unchanged percentages or an elevated proportion during fingolimod therapy (Kemmerer et al., 2020). Plasmablast percentages show different patterns or are unchanged during treatment with cladribine, interferon beta, dimethyl fumarate or glatiramer acetate. Further analyses by B cell repertoire mass sequencing or whole transcriptome analysis underlined that memory B cells are significantly affected during cladribine treatment (Ceronie et al., 2018; Rolfes et al., 2022; Ruschil et al., 2023) and also alemtuzumab treatment (Ruck et al., 2022). Data regarding changes in CSF immune cell subsets are limited and differences are difficult to assess due to the overall low number of immune cells. However, treatment with dimethyl fumarate, natalizumab, rituximab, ocrelizumab, and alemtuzumab resulted in reduced CSF B cell counts, whereas fingolimod did not alter the proportion of CSF B cells (Table 3). Plasmablasts were reduced during treatment with dimethyl fumarate, natalizumab and fingolimod (Table 3).

**TABLE 3:** B cell subsets changes in the cerebrospinal fluid (CSF) under various treatments.

	Total B cells	% Naive B cells	% Bmem	% Plasmablast	% DN	References
Dimethyl fumarate	↓	–	↔	↓	–	Høglund et al., 2018
Fingolimod	↔	(↑)	↔	(↓)	↔	Kowarik et al., 2021
Natalizumab	↓	↔	↓	↓	↔	Stüve et al., 2006; Warnke et al., 2015; Kowarik et al., 2021
Ocrelizumab	↓	–	–	–	–	Cross et al., 2019
Rituximab	↓	–	–	–	–	Monson et al., 2005; Cross et al., 2006
Alemtuzumab	↓	–	–	–	–	Müller-Miny et al., 2023

The arrows in parenthesis mean a slight change. DN, double negative.

In contrast to these approved treatments, other drugs that also affect B cells have been shown to be ineffective or even worsen MS. For example, atacicept was stopped in the ATAMS trial because of a pronounced conversion to MS in patients with optic neuritis. Although the exact mechanisms regarding B cells was not elucidated in the study, an increase in IL15 provided some evidence suggesting stimulation of memory B cells as a possible explanation for the clinical outcomes observed in the study (Kappos et al., 2014). In addition, the use of anti-TNF blockers such as infliximab, which can stimulate memory B cell activity, has been associated with an increased incidence of MS in patients with chronic disease (Avasarala et al., 2021). Regarding B cell depleting treatments, it is important to note that regulatory B cells may also be depleted, but this does not seem to drastically limit the therapeutic potential.

Interestingly, numerous MS treatments influence T cell function and T cell subset distribution (Martin et al., 2016), however, a direct effect on B cell populations seems to have an even more relevant effect on the disease course. The consistent effect of MS treatments on the memory B cell subset could further underpin this assumption due to their frequent occurrence in the CSF and their ability to recirculate into the periphery and to repeatedly participate in germinal center reactions. Of note, memory B cells are the primary site of persistent latent EBV infection which could partially explain the association between EBV infections and multiple sclerosis (Tracy et al., 2012).

## B cells in neuromyelitis optica spectrum disorder

### Anti-AQP4 antibody-secreting B cells as the major driver of NMOSD pathophysiology

Neuromyelitis optica spectrum disorder has been recognized as a separate disease entity with the discovery of autoantibodies against the water-channel aquaporin-4 (AQP4-AB) (Lennon et al., 2004). It could be clearly demonstrated that AQP4-AB bind to AQP4 channels on astrocytes triggering an activation of the complement cascade, with granulocyte, eosinophil, and lymphocyte infiltration, resulting in astrocyte damage. As a secondary event, oligodendrocyte injury leads to demyelination and neuronal loss (Carnero Contentti and Correale, 2021). Lineage analysis of AQP4- specific B cells from the peripheral blood and CSF B cells of NMOSD patients showed a clonal relationship with memory B cells, plasmablasts and DN B cells in the periphery during active disease. Immunoglobulin transcriptome analysis further indicated that expanded DN B cells undergo antigen-specific B cell maturation and are closely linked to AQP4-specific CSF B cells (Kowarik et al., 2017). Although it is believed that mis-priming and/or escape from tolerance mechanisms of peripheral B cells and the peripheral secretion of AQP4-AB might initiate NMOSD disease pathology, AQP-4 specific CSF plasmablasts have been shown to originate from peripheral B cells and intrathecally secrete AQP4-AB during active disease and thus might contribute to disease exacerbation (Kowarik et al., 2015).

### Double negative B cells in NMOSD—Link to rheumatic diseases

Besides the peripheral up-regulation and association of DN B cells and AQP4-reactive CSF plasmablasts in active NMOSD, DN B cells have received increasing attention in recent years, especially in SLE, where they have been found to be a marker of disease severity (Jenks et al., 2018; Szelinski et al., 2022). DN B cells are also elevated in the elderly, in infections and in other autoimmune diseases such as rheumatoid arthritis, Guillain-Barre syndrome and myasthenia gravis (Fraussen et al., 2019). DN B cells (CXCR5-) are extensively expanded in antibody-mediated autoimmune diseases such as SLE, where a worse disease course is correlated with an inflated population of DN B cells (CXCR5-), which are thought to represent plasmablasts precursors (Jenks et al., 2018; Szelinski et al., 2022). When co-cultured with Th cells, DN B cells have the capacity to differentiate into antibody-secreting cells (Janssen et al., 2020; Hoshino et al., 2022). Conversely, most of DN B cells in MS are not CXCR5-, indicating a different mechanism from that observed in NMOSD and SLE (Li et al., 2021a).

### Peripheral B cells and treatment: specific effects on B cell subsets in NMOSD

In the peripheral blood, plasmablasts (CD19intCD27highCD38highCD180-) have been shown to be up-regulated in NMOSD and secrete AQP4-AB following IL6 stimulation (Chihara et al., 2013). This dysregulatory shift toward antibody-secreting cells has been reaffirmed by different studies (Hoshino et al., 2022). As mentioned above, peripheral DN B cells have also been shown to be upregulated in the peripheral blood of NMOSD patients (Ruschil et al., 2020). Regulatory B cells are significantly reduced in AQP-4 positive patients compared to MS patients, possibly due to the high IL6 secretion, which subsequently inhibits the generation of regulatory B cells (Quan et al., 2013).

Most approved therapies currently target effector B cell lineages and the direct interaction caused by antibodies. B cell depletion, including the use of rituximab as an anti-CD20 antibody, as well as inebilizumab targeting CD19, has demonstrated effectiveness in treating NMOSD (Barreras et al., 2022; Nie and Blair, 2022). As DN B cells and plasmablasts lose CD20 expression, targeting the consistently expressed CD19 marker on both cell types may result in a more profound depletion and improve treatment effects (Agius et al., 2019). After receiving treatment with rituximab, the presence of regulatory B cells increases (Quan et al., 2015). Satralizumab and tocilizumab both inhibit the IL6 receptor, disrupting lymphocyte activation (Chu and Huang, 2022). Tocilizumab reduces memory B cells in the peripheral B cell subset, while regulatory B cells and plasmablasts remain unaffected (Traub et al., 2020). Eculizumab and ravulizumab are inhibitors of complement factor 5 and disrupt the complement signaling cascade initiated by anti-AQP4 antibodies. Eculizumab reduced the percentage of memory B cells in patients with myasthenia gravis (Li et al., 2021b).

The wide development of MS medications has led to experimental usage of these therapies in NMOSD in the past when no approved medications for NMOSD were available. Several medications have failed to show positive treatment effects or even worsened NMOSD disease course in single patients or small case series. Natalizumab and fingolimod appeared to increase the proportion of DN B cells in the periphery (Kemmerer et al., 2020), potentially clarifying why these drugs have not been shown to be effective in treating NMOSD. Along these lines, paradoxical rebound under rituximab therapy in NMOSD patients may be explained by an increase of CD20-negative DN B cell/plasmablasts and an asynchronous B cell depletion (Kowarik et al., 2017). Other approved MS drugs that were not effective or even harmful when used in single NMOSD cases included alemtuzumab, dimethyl fumarate, glatiramer acetate, interferon-β, fingolimod and natalizumab. Analyses of peripheral B cell subsets reveals that the mentioned medications might increase the proportion of plasmablasts, as wells as B cells supporting the pathophysiology of NMOSD. Some medications also increase serum interleukin-6 levels and serum BAFF levels, which could contribute to the pro-inflammatory reaction, worsening the disease course (Traub et al., 2020).

## Discussion

Several lines of evidence suggest that there are significant differences in the composition of peripheral B cells between MS and NMOSD. Whereas only minor changes in peripheral B cell subsets are observable in untreated MS patients, alterations and an up-regulation of DN B cells and plasmablasts are apparent in NMOSD. Although it is possible to effectively treat both diseases with B cell-depleting therapies that broadly target circulating B cells, distinct treatment effects on particular B cell subsets can be observed in both diseases. Numerous MS treatments have demonstrated effective targeting of memory B cells, suggesting a significant pathophysiological role in MS. Vice versa, this assumption is underlined by the inefficiency of therapies that potentially increase peripheral memory B cell activity. In NMOSD, efficient treatments have been shown to target the stimulation of effector B cells such as plasmablasts or deplete effector B cells including DN B cells. In this context, anti-CD19 depletion might be even more effective than anti-CD20 depletion since DN B cells and plasmablasts show a low frequency or even lack the CD20 surface marker. The ineffectiveness of several MS drugs in treating NMOSD most likely results from their failure to target effector B cells or to increase the proportion of DN B cells and plasmablasts. Based on these results the pathophysiological role of B cells has to be discussed in both diseases. While these treatment effects highlight the role of anti-AQP4 antibody-secreting B cells in NMOSD, several MS treatments have profound effects on the peripheral memory B cell subset and reduce CSF B cell numbers. The widespread inconsistency regarding clear B cell targets in MS raises the question of whether the primary pathophysiological role of B cells in MS is indeed autoantibody production. Instead, there is evidence suggesting that memory B cells can act as antigen-presenting cells (Figure 1), possibly supporting autoimmune circuits and the activation of autoreactive T cells (Morbach et al., 2011; Rastogi et al., 2022). Since CSF B cells are able to re-circulate from the CNS to the periphery (Ruschil et al., 2021), subsets of peripheral memory B cells could possibly present CNS related antigens they have once encountered within the CNS (during an acute relapse). The persistence of EBV in memory B cells could possibly alter memory B cell functions and persistence (Tracy et al., 2012). In addition, alterations in peripheral tolerance (Pugliese, 2004) and the association between MS and certain HLA class II alleles of the DR2 haplotype, which might influence antigen presentation, could further substantiate this hypothesis. Although cases of onset or exacerbation of NMOSD following EBV and other pathogens have been reported, no clear association between NMOSD and a specific virus has been found (Frau et al., 2023); however, environmental factors cannot be ruled out. In this context, disruption of central tolerance mechanisms is likely to be a critical factor in the development of NMOSD, with poly reactive naive B cells possibly transforming into antibody-producing cells (Kinnunen et al., 2013). Similar to NMOSD, patients with antibodies against myelin oligodendrocyte glycoprotein (MOG) appear to share pathophysiologic mechanisms that remain to be fully elucidated (Lana-Peixoto and Talim, 2019). To date, no approved treatments are currently available for MOG antibody associated diseases.

In conclusion, this review highlights distinct differences in the pathophysiology of B cells in MS and NMOSD, as revealed by the analysis of peripheral and CSF B cell subsets in untreated patients and treatment-related effects of different drugs. Although further studies are needed to fully understand the exact triggers of autoimmunity and development of pathologic B cell subsets in both diseases, current knowledge suggests more refined treatment strategies targeting defined B cell subsets rather than deep B cell depletion. Bruton’s tyrosine kinase inhibitors are an interesting new treatment approach targeting B cells, but their effects and exact role on B cell subsets remain to be determined. Specific targeting of memory B cells in multiple sclerosis vs. antibody-secreting B cells, including the DN B cell subsets, in NMOSD may be promising treatment strategies in the near future.

## Author contributions

MT: Conceptualization, Writing – original draft, Writing – review and editing, Formal Analysis, Investigation, Resources. NV: Data curation, Resources, Visualization, Writing – review and editing. CR: Investigation, Supervision, Validation, Writing – review and editing. MK: Conceptualization, Investigation, Methodology, Supervision, Validation, Writing – review and editing.
